# Neutrophil-to-Lymphocyte Ratio Adds Valuable Information Regarding the Presence of DKA in Children with New-Onset T1DM

**DOI:** 10.3390/jcm12010221

**Published:** 2022-12-28

**Authors:** Alexandra-Cristina Scutca, Delia-Maria Nicoară, Monica Mărăzan, Giorgiana-Flavia Brad, Otilia Mărginean

**Affiliations:** 1 Department of Pediatrics, University of Medicine and Pharmacy “Victor Babes”, 300040 Timisoara, Romania; 2 Department of Pediatrics I, Children’s Emergency Hospital “Louis Turcanu”, 300011 Timisoara, Romania; 3 Department XI Pediatrics, Discipline I Pediatrics, Disturbances of Growth and Development in Children–BELIVE, 300011 Timisoara, Romania

**Keywords:** new-onset T1DM, diabetic ketoacidosis, children, NLR score

## Abstract

Diabetic ketoacidosis (DKA) is an acute life-threatening complication occurring mainly at the onset of type 1 diabetes mellitus. The neutrophil-to-lymphocyte ratio (NLR), a marker for systemic inflammation, has recently generated increasing interest in many chronic diseases. The aim of this cross-sectional study was to determine the value of the neutrophil-to-lymphocyte ratio (NLR) in association with DKA severity across these cases. A total of 155 children with new-onset type 1 DM from one large center were included in the study. Total and differential leukocyte counts were measured upon admission and calculation of the NLR was performed. Patients were classified into four groups: without DKA, mild, moderate, and severe DKA at disease onset. Total WBCs, neutrophils, and monocytes increased with DKA severity (*p*-value < 0.005), while eosinophiles displayed an inverse relationship (*p*-value < 0.001). Median NLR scores increased from those without ketoacidosis (1.11) to mild (1.58), moderate (3.71), and severe (5.77) ketoacidosis groups. The statistical threshold value of the NLR in predicting DKA was 1.84, with a sensitivity of 80.2% and a specificity of 80%. Study findings indicate that a higher NLR score adds valuable information regarding the presence of DKA in children with new-onset T1DM.

## 1. Introduction

Diabetic ketoacidosis (DKA) is an acute life-threatening complication occurring mainly at the onset of type 1 diabetes mellitus [1,2], with an incidence rate that spans from 13 to 80% [3,4,5]. Being a form of systemic inflammatory state [6,7], inflammatory markers such as blood leukocytes and C-reactive protein (CRP) play a key role in the pathogenesis [8,9]. Although complete blood counts (CBCs) are a part of the routine evaluation in diabetic patients, white blood cell (WBC) fractions did not receive significant attention from diabetes specialists in the past [10]. In recent years, however, there has been growing interest regarding the neutrophil-to-lymphocyte ratio (NLR) as a marker of systemic inflammation in cardiac diseases, neoplasms, and obesity, as well as in diabetes-related complications such as diabetic foot ulcers and retinopathy [11,12,13,14]. Against this background, our aim was to study the association between the NLR and DKA severity among children with new-onset T1DM.

## 2. Materials and Methods

### 2.1. Patient Recruitment

This cross-sectional study included data from one of the largest Romanian reference centers for pediatric T1DM. We reviewed 181 consecutive T1DM patients charts from the Pediatric Emergency Hospital “Louis Turcanu” in Timisoara, Romania, between 1 January 2015 to 30 June 2022, in accordance with the principles of the Declaration of Helsinki (1975, revised in 2013). Ethical approval was obtained from the ethics committee.

Inclusion criteria for cases were noted as new-onset T1DM in children aged 0 to 18 years, with or without diabetic ketoacidosis. Diagnosis of type 1 DM was established according to the American Diabetes Association (ADA) criteria of 2021. Exclusion criteria were as follows: infectious states, any other medical conditions that could alter hematological parameters, and patients with other types of diabetes.

Patients with DKA had a plasma glucose level > 11 mmol/L, a urine ketone level defined as moderate to high (+ to +++), and an arterial pH value < 7.30 at the time of admission. The ADA (American Diabetes Association) criteria for DKA severity were used: mild DKA, 7.20 ≤ pH < 7.30; moderate DKA, 7.10 ≤ pH< 7.20; and severe DKA, pH < 7.10 [15].

### 2.2. Biochemical Assays

Laboratory tests, including routine biochemistry tests and arterial gas analysis, were performed in the hospital laboratory. Blood samples were drawn at admission before the initial therapy, to avoid posttreatment changes in CBC parameters, and collected for differential WBC counts in tubes with EDTA and processed using a Sysmex XN-550 (Sysmex Corporation, Kobe, Japan) automatic blood counting system. Glycated Hb (HbA1c) was measured using a high-performance liquid chromatography kit supplied by Cobas E 411–Roche, Japan. Peptide C was evaluated using automated chemiluminescent assay (Cobas E 411–Roche, Tokyo, Japan). Neutrophil-to-lymphocyte ratios (NLR) were calculated.

### 2.3. Statistical Analysis

All data analysis was performed using the standard computer program Statistical Package for the Social Sciences (SPSS) for Windows, version 28 (SPSS Inc., Chicago, IL, USA) and GraphPad Prism9. The Shapiro–Wilk test was used to test the normality of the data distribution. Normally distributed variables were expressed as mean ± standard deviation (SD) and non-normally distributed variables were expressed as medians with interquartile ranges. Intergroup comparisons were performed by using an independent-sample t test and one-way ANOVA for normally distributed continuous data and Chi-Square tests for categorical variables. Non-normally distributed data were compared among multiple groups using the Kruskal–Wallis test. GraphPad Prism version 9 was used for univariate analysis with a post hoc procedure regarding NLR scores in DKA patients. Multiple regression analysis was performed to evaluate the association between the NLR or WBC parameters and the occurrence of DKA in T1DM patients. Receiver operating characteristic (ROC) curve analysis was plotted to compare the discrimination performance of HbA1c, C peptide, and CBC parameters in predicting DKA severity. The optimal threshold values were obtained using Youden’s index (sensitivity + specificity − 1, ranging from 0 to 1) and the maximized area under the curve (AUC). A *p* value (two-tailed) < 0.05 was considered statistically significant.

## 3. Results

### 3.1. Patient Characteristics Stratified by DKA Grade

Following the retrospective revision of T1DM electronic charts, there were 181 newly diagnosticated children. We excluded 26 patients due to concomitant acute infections. The study group included 155 children (76 males, 79 females), with a mean age of 9.00 ± 4.39 years (range 0–18 years).

According to the onset characteristics, fasting blood glucose, islet autoantibodies, serum C-peptide, ketone bodies, and blood gas analysis results [16], children with new-onset T1DM were divided into four groups: the non-DKA (*n* = 35), mild DKA (*n* = 25), moderate DKA (*n* = 33), and severe DKA (*n* = 62) group.

There were no significant differences in terms of age among the four groups. Regarding gender, there were more female patients in the severe DKA group. HbA1c levels were approximately equal in the four groups (mean = 11.40 ± 2.01).

### 3.2. Differential WBC Counts

As shown in Table 1, there was a significant difference in the total and differential WBC counts regarding the four groups, especially regarding total WBCs, neutrophils, and monocytes which increased with DKA severity (*p* < 0.0005). Eosinophiles displayed an inverse relationship to DKA severity (*p*-value < 0.001), decreasing with DKA severity. Lymphocytes were statistically lower in severe DKA patients compared to those with mild and moderate DKA.

### 3.3. NLR Score

**Figure 1:**
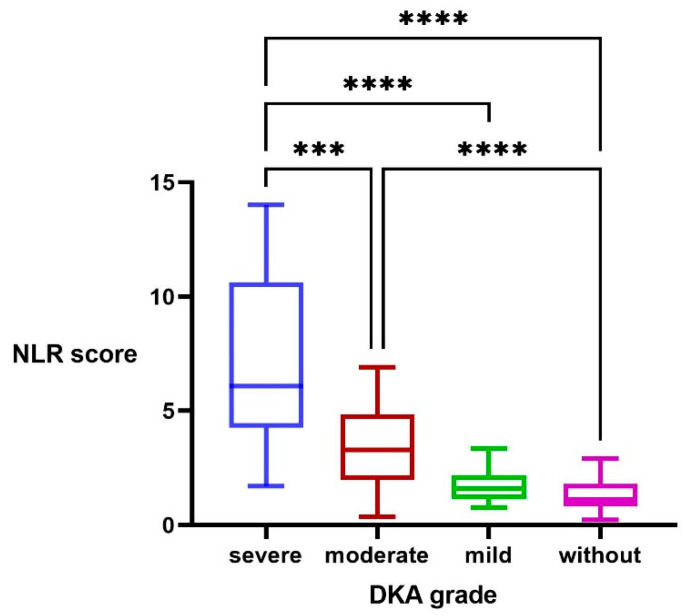
Univariate analysis with post hoc procedure regarding NLR scores in DKA patients; **** *p* = 0.000, *** *p* = 0.002.

A Kruskal–Wallis H test was performed to determine if there were significant differences in NLR scores between children without ketoacidosis and those with mild, moderate, or severe ketoacidosis. The distributions of NLR scores were not similar for all groups, as assessed by visual inspection of a boxplot. Median NLR scores increased from those without ketoacidosis (1.11) to mild (1.58), moderate (3.71), and severe (5.77) ketoacidosis groups (Figure 1). The distributions of NLR scores were significantly different between groups: X^2^(3) = 97.681, *p* = 0.000. Subsequently, multiple comparisons were performed through post hoc analysis using Dunn’s (1964) procedure with a Bonferroni correction. Adjusted *p*-values are presented. This post hoc analysis revealed statistically significant differences in median NLR scores between those with severe DKA and those with moderate (*p* = 0.002), mild (*p* = 0.000), or no DKA (*p* = 0.000); between mild and moderate DKA (*p* = 0.012), but not between those with mild DKA and no DKA (*p* = 1.000).

### 3.4. Correlation and Regression Analyses

A multiple regression analysis was performed to determine the correlation between blood pH and age, gender, HbA1c, C peptide, and NLR. The multiple regression model was statistically associated with blood pH: *F*(5, 130) = 41.485, *p* < 0.001, adj. *R*^2^ = 0.600. NLR score and age added significantly to the association, *p* < 0.001. Regression coefficients and standard errors are listed in Table 2.

### 3.5. Receiver Operating Characteristics (ROC) Curve Analysis

**Figure 2:**
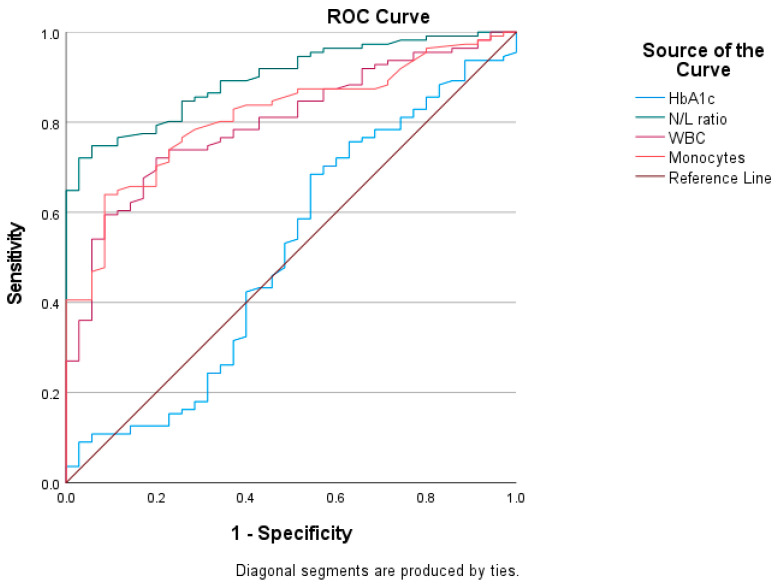
ROC curve analysis of HbA1c, WBCs, monocytes, and NLR; ROC, receiver operating characteristic. Significant differences were found (*p* < 0.000, respectively, for NLR, WBCs, and monocytes).

**Figure 3:**
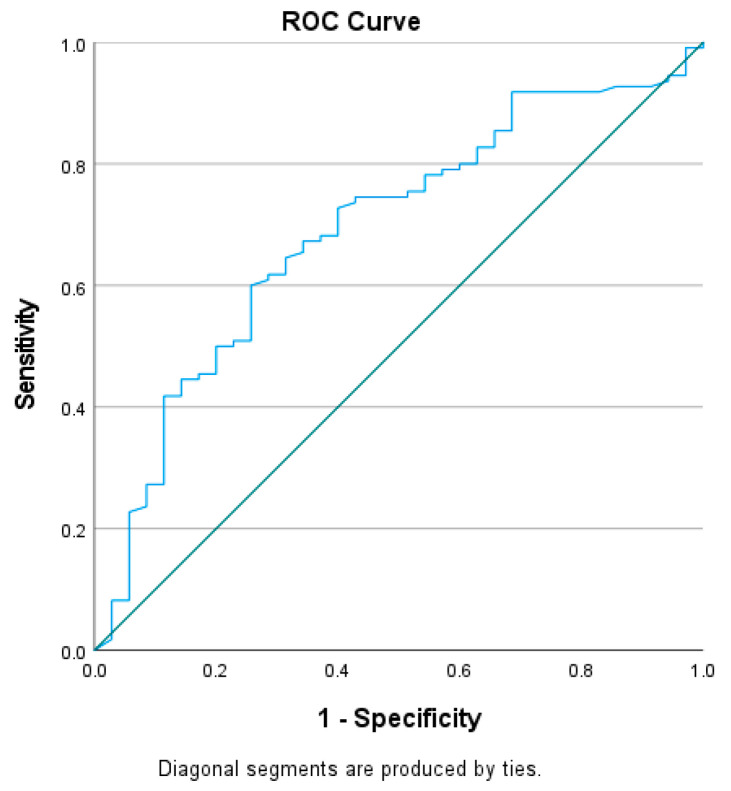
ROC curve analysis of C peptide; ROC, receiver operating characteristic.

The diagnostic ability of HbA1c, C peptide, WBCs, monocytes, and NLR in predicting DKA was analyzed by the ROC curve (Figure 2 and Figure 3). The AUCs and cut-off values were calculated according to their specificity and sensitivity as predictive factors. The most influential indicators for DKA patients were WBCs (AUC 0.800; 95% CI: 0.723–0.877, *p* < 0.000), monocytes (AUC 0.815; 95% CI: 0.742–0.887, *p* < 0.000), NLR (AUC = 0.903; 95% CI: 0.854–0.952, *p* < 0.000), and, to a lesser extent, C peptide (AUC = 0.690; 95% CI: 0.591–0.789, *p* = 0.001), as opposed to HbA1c (Table 2).

The statistical threshold value of the NLR in predicting DKA was 1.84, with a sensitivity of 80.2% and a specificity of 80% (Table 3).

## 4. Discussion

Type 1 diabetes mellitus (T1DM) represents one of the most frequent chronic illnesses affecting children [17]. Previous studies [16,17,18,19,20,21,22,23] have indicated an increase in both the frequency and severity of DKA cases in recent years. In our research, 81% of cases with T1DM presented with DKA, almost half of which were severe.

WBC counts, fractions, and indices, among which the NLR has received attention in recent years, were correlated with inflammation-associated diseases such as systemic hypertension [24], intracranial atherosclerosis [25], neoplasia [26], obesity [14], and type 2 diabetes [27,28,29].

The shifts in the percentage formula of white blood cells (increase in total WBCs, neutrophils, and monocytes; decrease in lymphocytes and eosinophiles) were similar to those cited in the literature [17,30].

Aside from systemic inflammation [31,32,33], the NLR, a well-characterized systemic inflammatory response marker [34], can also reflect both innate and adaptive immune (dys)function [9,35,36]. This simple ratio, which combines the predictive power of both increased neutrophil and decreased lymphocyte counts, has the advantage of being ubiquitous, cost effective, and also more stable compared with the absolute count [9,30,37]. Results from the present study are consistent with previous publications [10,30,38], in that WBC count and the NLR were found to be higher in patients with DKA.

Median NLR scores in our case were significantly different between groups, increasing from those without ketoacidosis (1.11; 0.80–1.80) to mild (1.58; 1.17–1.93), moderate (3.71; 1.98–4.85), and severe (5.77; 4.04–9.63) ketoacidosis groups. Our results regarding pediatric patients are consistent with a previous study addressing adults with DKA, which regards the NLR as a possible marker of the underlying severity of acute systemic inflammation in uninfected DKA patients [6]. Aside from the obvious effect of hemoconcentration on the NLR, the potential relationship between hyperglycemia and an increased NLR has been addressed in previous studies [39]. One possible explanation is that WBCs that are activated by advanced glycation end-products produce pro-inflammatory cytokines [29]. However, our study did not reveal statistical differences among the four groups in terms of mean HbA1c levels. This is consistent with some studies regarding children with DKA [40,41,42], and in opposition with other studies [17]. Another explanation is the fact that, in DKA, acute hyperglycemia promotes the accumulation of reactive oxygen species (ROS) which can damage peripheral blood lymphocytes’ DNA. This in turn may cause the apoptosis of lymphocytes and affect their proliferation [6,43,44].

In the present study, with new-onset T1DM children grouped according to blood pH, multivariate logistic regression analysis was performed in order to assess whether confounding exists between age, sex, HbA1c, C peptide, and NLR regarding blood pH. The NLR displayed a good discriminatory power regarding association with DKA, through correlation with blood pH.; age at onset, and, to a lesser extent, C peptide added statistically significantly to the prediction. This is consistent with a previous published study regarding adult T1DM patients [10] but, to our knowledge, was not yet reported in children. An upside to examining children is their lack of many confounding factors that can affect NLR levels, such as common medications and comorbidities present in adult patients with diabetes.

Assessing the ROC curve, the presence of DKA in our study lot was associated with an elevated NLR, monocytes, and WBCs. The area under the curve was largest for the NLR, with values above 1.84 being most frequently present in children with DKA (sensitivity of 80.2% and specificity of 80%). Regarding C peptide, plasma values were negatively correlated with the presence of DKA, mainly values below 0.690 ng/mL (sensitivity of 68.2% and specificity of 60%).

There were some limitations in the present study. Firstly, the sample size was relatively small, which could limit the power of the analyses. Secondly, our patients are only from one hospital, so that selection bias cannot be ruled out. Additionally, only one measurement of CBC and subsequent NLR calculation were used in the analysis: those upon admission. As such, there was no monitoring of the dynamic trend of the NLR. We look forward to additional multicenter studies with large samples.

## 5. Conclusions

This study adds complementary laboratory data regarding children with DKA at onset of T1DM [10,45]. It underlines the fact that higher NLR levels were associated with an increased prevalence of DKA in children with new-onset T1DM, and positively correlated with the DKA grade.

To the authors’ knowledge, it represents the first study to evaluate the NLR based on DKA severity in children with new-onset T1DM. This finding has clinical significance, especially in pre-hospital settings, where blood gas analysis is usually not part of routine investigations, because it may improve the early diagnosis of DKA in children with elevated glucose level and thereby facilitate proper care.

## Figures and Tables

**Table 1:** Demographic data and laboratory findings of all patients.

Parameters	Non-DKA (*n* = 35)	Mild DKA (*n* = 25)	Moderate DKA (*n* = 33)	Severe DKA (*n* = 62)	*p*
Age (years)	10 (5–13)	9 (6.5–13)	7.00 (3.50–11)	9.00 (5–12)	0.381
Males% (*n*)	42 (15)	76 (19)	48 (16)	41 (26) ^b^	**0.028**
HbA1c (%)	11.37 ± 1.95	11.68 ± 1.94	11.37 ± 2.16	11.52 ± 2.04	0.931
C-peptide (ng/mL)	0.639 (0.41–0.94)	0.481 (0.35–0.67)	0.533 (0.29–0.77)	0.330 (0.18–0.47) ^a, c^	**<0.001**
Blood pH	7.36 (7.34–7.37)	7.28 (7.23–7.29)	7.17 (7.13–7.20) ^a^	6.97 (6.89–7.03) ^a, b, c^	**0.000**
WBCs (×10^3^/mm^3^)	8.53 (6.64–10.13)	8.12 (6.68–8.90)	12.27 (9.92–15.47) ^a, b^	18.78 (14.06–24.52) ^a, b, c^	**<0.001**
Neutrophils (×10^3^/mm^3^)	3.79 (2.99–5.24)	4.58 (3.38–5.21)	8.97 (6.24–12.6) ^a, b^	14.63 (11.06–18) ^a, b, c^	**0.000**
Lymphocytes (×10^3^/mm^3^)	2.92 (2.50–4.66)	2.71 (1.96–3.49)	2.86 (2.04–3.96)	2.33 (1.59–3.2) ^a^	**0.003**
Thrombocytes (×10^3^/mm^3^)	299 (229–327)	259 (223–344)	347 (283–405) ^a^	342 (388–422) ^a, b^	**<0.001**
Monocytes (×10^3^/mm^3^)	0.60 (0.49–0.74)	0.68 (0.51–0.77)	0.87 (0.70–1.39) ^a^	1.71 (1.15–2.40) ^a, b, c^	**<0.001**
Eosinophiles (×10^3^/mm^3^)	0.12 (0.05–0.22)	0.09 (0.03–0.13)	0.06 (0.02–0.20)	0.00 (0–0.02) ^a, b^	**<0.001**
NLR	1.11 (0.80–1.80)	1.58 (1.17–1.93)	3.71 (1.98–4.85) ^a, b^	5.77 (4.04–9.63) ^a, b, c^	**0.000**

One-way ANOVA, Kruskal–Wallis H-test, and Chi-Square test. Data are expressed as mean ± standard deviation, median (interquartile range, IQR) or percentage (*n*, %). ICU, Intensive Care Unit; HbA1c, glycated hemoglobin; WBC, white blood cell count; NLR, neutrophil-to-lymphocyte ratio. Statistically significant differences, with a probability value of *p* < 0.05, are represented in bold. Compared with the non-DKA group, ^a^ *p* < 0.05. Compared with the mild DKA group, ^b^ *p* < 0.05. Compared with the moderate DKA group, ^c^ *p* < 0.05.

**Table 2:** Linear regression analysis of factors related to blood pH in new-onset T1DM patients.

pH	B	95% CI for B		SE B	ß	*R* ^2^	∆*R*^2^
		LL	UL				
Model						0.654	0.640
Age	0.010 ***	0.004	0.015	0.003	0.239 ***		
Gender	0.003	−0.036	0.042	0.020	0.007		
HbA1c	0.001	−0.009	0.011	0.005	0.008		
C peptide	0.074 *	−0.014	0.135	0.030	0.145 *		
NLR	−0.038 ***	−0.044	−0.032	0.003	−0.770 ***		

B = unstandardized regression coefficient; CI = confidence interval; LL = lower limit; UL = upper limit; SE B = standard error of the coefficient; ß = standardized coefficient; *R*^2^ = coefficient of determination; ∆*R*^2^ = adjusted *R*^2^. * *p*< 0.05, *** *p* < 0.001.

**Table 3:** ROC curve area and cut-off Values for predicting DKA. AUC = area under the curve; S.E. = standard error; CI = confidence interval.

Variable	AUC	S.E.	95% CI	Cut-Off	Sensitivity %	Specificity %
HbA1c	0.504	0.060	0.386–0.622	11.38	49.5	51.4
C peptide	0.690	0.050	0.591–0.789	0.554	68.2	60.0
WBC	0.800	0.039	0.723–0.877	8.860	79.2	57.1
Monocytes	0.815	0.037	0.742–0.887	0.675	80.2	62.9
NLR	0.903	0.051	0.854–0.952	1.84	80.2	80.0

## Data Availability

The data are not publicly available due to reasons of privacy.
